# Enhanced Biomass Yield of and Saccharification in Transgenic Tobacco Over-Expressing β-Glucosidase

**DOI:** 10.3390/biom10050806

**Published:** 2020-05-23

**Authors:** Eun Jin Cho, Quynh Anh Nguyen, Yoon Gyo Lee, Younho Song, Bok Jae Park, Hyeun-Jong Bae

**Affiliations:** 1 Bio-Energy Research Center, Chonnam National University, Gwangju 61186, Korea; choej47@gmail.com (E.J.C.); quynhanhnqhc@gmail.com (Q.A.N.); jagnad@naver.com (Y.S.); 2 Department of Bioenergy science and Technology, Chonnam National University, Gwangju 61186, Korea; spake123@naver.com; 3 Division of Business and Commerce, Chonnam National University, Yeosu 500-749, Korea; bjpark73@jnu.ac.kr

**Keywords:** β-glucosidase, transgenic tobacco plant, biomass yield, overexpression, saccharification

## Abstract

Here, we report an increase in biomass yield and saccharification in transgenic tobacco plants (*Nicotiana tabacum* *L*.) overexpressing thermostable β-glucosidase from *Thermotoga maritima*, *BglB*, targeted to the chloroplasts and vacuoles. The transgenic tobacco plants showed phenotypic characteristics that were significantly different from those of the wild-type plants. The biomass yield and life cycle (from germination to flowering and harvest) of the transgenic tobacco plants overexpressing *BglB* were 52% higher and 36% shorter than those of the wild-type tobacco plants, respectively, indicating a change in the genome transcription levels in the transgenic tobacco plants. Saccharification in biomass samples from the transgenic tobacco plants was 92% higher than that in biomass samples from the wild-type tobacco plants. The transgenic tobacco plants required a total investment (US$/year) corresponding to 52.9% of that required for the wild-type tobacco plants, but the total biomass yield (kg/year) of the transgenic tobacco plants was 43% higher than that of the wild-type tobacco plants. This approach could be applied to other plants to increase biomass yields and overproduce β-glucosidase for lignocellulose conversion.

## 1. Introduction

Lignocellulosic biomass, particularly originating from crops, is abundant and available globally. It acts as an excellent source material for the production of biofuels and value-added products [1,2]. In order to develop biorefineries for processing lignocellulosic biomass, several challenges should be overcome, especially reducing enzyme costs and improving lignocellulosic materials to ensure that they can be hydrolyzed more easily. Recently, several studies focusing on plant genetic engineering have aimed to improve the feasibility of biofuel production by reducing the recalcitrant components of plant biomass and increasing the heterologous overexpression of enzymes for the autohydrolysis of cellulose in plants, in order to improve conversion yield [3,4,5]. Another approach is to increase the production of biomass via plant genetic engineering [6,7]. A recent study reported a considerable improvement in biomass production by fixing photosynthetic inefficiencies, making transgenic tobacco plants to more efficiently recaptures the by-products of photosynthesis with less energy loss, leading to approximately 40% higher biomass production than that of wild-type tobacco plants [8]. However, despite some successes, practical application of this technique has not been confirmed.

β-glucosidases belong to a large family of glycoside hydrolases (http://www.cazy.org/fam/GH1.html) and catalyze the hydrolysis of the β-glucosidic bond between two carbohydrate moieties, or between a carbohydrate and an aglucone moiety. In plants, β-glucosidases play important roles in growth and development, including the (1) release of active forms of phytohormones from their inactivated forms (conjugated-phytohormones), (2) formation of intermediates in cell wall lignification, (3) degradation of cell wall of the endosperm during germination, and (4) activation of chemical defense compounds [9,10,11,12]. Recently, β-glucosidases have garnered attention owing to their biotechnological and industrial applications. In particular, several studies on the heterologous overexpression of β-glucosidase genes in plants have reported an interesting phenomenon, that is, the overexpression of β-glucosidases genes results in enhanced growth and development, increased biomass biosynthesis, and improved saccharification. These changes including early flowering and increased biomass, height, internode length, and leaf area, depending on the changes in hormone levels in transgenic plants have been attributed to the hydrolyzing functions of β-glucosidases, which release activated forms of phytohormones from their inactivated forms (conjugated hormones) [6,7,9,13]. However, the underlying mechanisms in the transgenic plants remain unclear, particularly those involved in changes in genome transcription levels [14,15,16]. In contrast, there were no changes in the level of hormones in transgenic plants in a previous study [17], suggesting that the earlier explanations based on changes in phytohormone levels do not fully explain the observed changes in transgenic plants. Therefore, studies that aim to evaluate the mechanisms leading to pronounced changes in transgenic plants are necessary.

Increased saccharification has been reported in transgenic plants overproducing β-glucosidases, and this can be explained by changes in biomass structures due to modifications in the chemical composition (lignin and saccharide content) of transgenic plants compared with those of the control [18,19,20]. However, the mechanisms underlying increased saccharification, especially how a transgenic plant develops after these changes in the biomass structure or how it achieves enhanced biomass production in some cases, as mentioned above, are not completely clear. Our previous studies indicated that thermostable β-glucosidase from *Thermotoga maritima* (*BglB*) could be targeted to the organelles (chloroplasts and vacuoles) of transgenic tobacco plants, while retaining its biological function of enzymatic hydrolysis of lignocellulosic biomass. Furthermore, we provided evidence of enhanced growth and development of transgenic tobacco plants [7,21]. As our previous studies produced encouraging results, in this study, we conducted experiments using many generations of transgenic tobacco plants, with *BglB* targeted to the chloroplasts and vacuoles, to confirm that the pronounced phenotypic changes in transgenic tobacco plants are stable and can be inherited through generations, and that *BglB* can be successfully produced by tobacco plants. The results of our RNA microarray analysis provide clear evidence of the changes in genome transcription levels in transgenic tobacco plants in comparison with those in wild-type tobacco plants. Thus, our study provides a novel explanation for enhanced phenotype in transgenic tobacco plants. Because the transgenic tobacco plants showed a significant increase in biomass production, a techno–economic assessment was conducted to confirm the advantages of the *BglB* transgenic tobacco plants. Moreover, expressing *BglB* in transgenic tobacco plants may be beneficial because such plants have a superior conversion efficiency of lignocellulosic biomass, because of the combination of β-glucosidases with cellulases. Here, we demonstrate that *BglB* transgenic plants might be suitable for multi-target biorefinery processes, as their beneficial qualities include an increase in lignocellulosic enzymes. The use of biomass from transgenic plants in biorefinery processes is likely to ensure a high enzymatic conversion yield.

## 2. Materials and Methods

### 2.1. Collection of Transgenic Tobacco Plants Overexpressing Thermostable β-Glucosidase T. maritima BglB Targeting Chloroplasts and Vacuoles, Planting Conditions, and Measurement of Phenotypic Characteristics

Seeds of wild-type tobacco plants (*Nicotiana tabacum L*.) were sprayed in MS medium (Murashige and Skoog medium), while seeds of many lines of the T1 generation of transgenic tobacco plants overexpressing thermostable β-glucosidase *T. maritima BglB* targeting chloroplasts and vacuoles, originating from our previous studies [7,21], were sprayed in MS medium containing 100 mg/L kanamycin. Both seedlings types were grown in a growth chamber under a 16-/8-h light/dark cycle at 25 ± 3 °C, and the day of germination were noted. After 2 weeks, seedlings were used to determine β-glucosidase activities. Based on these results, we selected three transgenic lines from the chloroplast- and vacuole-targeted transgenic tobacco plants having the highest β-glucosidase activities, with at least 30 plants/lines, and moved them to a greenhouse (in 10-L pots) in soil-perlite mixtures at 25 ± 3 °C under a 16-/8-h light/dark photoperiod and a light intensity of 100 mmol m^−2^s^−1^ for development. Phenotypic characteristics appearing during plants growth and development, including leaves area, stem height, number of fruits, fresh/dry weight (FW/DW) of fruits, stem, leaves, roots, and total plants, as well as flowering time (days after germination, DAG), were measured. Flowers were covered before flowering to prevent crossbreeding in post generations, and seeds were harvested carefully for further experiments.

### 2.2. Measurement of β-Glucosidase Enzymatic Activities

Measurement of β-glucosidase *BglB* activities were followed by previous study [7]. Briefly: samples from fresh materials (seedlings, leaves, stems, or roots) and dried materials (for saccharification) of wild-type tobacco plants and T1 to T4 generation transgenic tobacco plants were ground to a powder in liquid nitrogen. Then, the powder was suspended in protein extraction buffer at pH 8.0 (50 mM Tris–HCl, 5 mM Na_2_EDTA, 20 mM Na_2_S_2_O_5_ × 5H_2_O, 100 mM KCl, 5% glycerol, 1% β-mercaptoethanol). Debris was removed by centrifugation at 13,000× *g* for 20 min at 4 °C. Total soluble protein (TSP) in the supernatants was measured using the Bradford method, and an amount of extracted protein equivalent to 10 μg of TSP was used to examine β-glucosidase *BglB* enzymatic activity using p-nitrophenyl b-d-glucopyranoside (p-NPG) as the substrate. One unit of β-glucosidase is defined as the amount of enzyme that releases 1 mmol of p-nitrophenol (p-NP) from the p-NPG substrate under the conditions of the assay: a mixture containing 10 mM p-NPG in citrate–phosphate buffer (pH 4.5) was incubated with the enzyme for 30 min at 70 °C in a total volume of 1 mL. The reaction was stopped by adding 1 M Na_2_CO_3_, and absorbance was measured at 405 nm.

### 2.3. mRNA Microarray and Transcriptional Expression Levels

A mRNA microarray experiment was conducted as follows: wild-type and T1 generation of transgenic tobacco plants ChB-1 line were grown in a greenhouse at 25 ± 3 °C under a 16-/8-h light/dark photoperiod and a light intensity of 100 mmol m^−2^s^−1^. The fifth and sixth leaves (from the top) were collected and kept in liquid nitrogen. Total RNA was extracted following the previous study [7], and cDNA was synthesized using avian myeloblastosis virus (AMV) reverse transcriptase (Promega, San Luis Obispo, CA, USA) with random hexamers. The cDNA libraries were then transferred to Genotech Ltd. Co. (Daejeon, Republic of Korea) for microarray analysis. Briefly, cDNA library clones including expressed sequence tags (ESTs) with short, single pass sequence reads derived from cDNA libraries of wild-type and transgenic tobacco plants ChB-1 lines were single pass sequenced from the 5′ end using the M12 primer, collated, and trimmed for vector sequences. Then, ESTs were assembled into a UniGene set with specific parameters. The sequences were filtered for polyA tails, low complexity, low quality, and short sequences. The UniGene set was annotated using BLASTX against a database of protein sequences from *Arabidopsis thaliana* (The Arabidopsis Information Resource [TAIR] 10.0 release).

A RT-PCR experiment was conducted to check transcriptional expression levels of high-mobility group (HMG)-Y-related protein A-like, acetyltransferase F-box protein, and malate dehydrogenase (MDH) [NADP] chloroplastic-like. Total RNA was extracted from the leaf tissues, cDNA library was synthesized with random hexamers, and the RT-PCR was performed using primer sets: HMG-Y-related protein A-like—FP-ATG GCT ACT GAA TTT GTC AAC AAG, RP- TTA CTC AAC AAC ACC ATT TTG TGC TGA; acetyltransferase F-box protein—FP-ATG ATC ATT ACT AAG CAG TAT CGC, RP- TCA TGC ATC ACT GTG ATC TGA AGC; and MDH [NADP], chloroplastic-like—FP-ATG GCA GCA ACA TCA GCA ACT ACT, RP-CTA AGC AGC TAC AGG TTC CTT CTG.

### 2.4. Carbohydrate Contents and Composition Analysis

Qualitative analysis of the monosaccharide (MS) compositions of biomass from each part of the wild-type and transgenic tobacco plants, before and after pretreatment, was performed as following previous study [22]. Briefly, carbohydrates of each part (leaves, stem, and roots) of tobacco plants and biomass samples for saccharification were determined through the two-step acid hydrolysis procedure of the National Renewable Energy Laboratory TP-510-42618 [23]. Concentrations of MSs were measured by high performance liquid chromatography (HPLC) (Waters 2695 system, MA, USA) with an Aminex HPX-87P column (300 × 7.8 mm, Bio-Rad, Hercules, CA, USA) and a refractive index detector (Waters 2414 system). The analysis was performed with 5 mM H_2_SO_4_ as a mobile phase at an isocratic flow rate of 0.6 mL/min.

### 2.5. Pretreatment by Mild Acid and Saccharification

Pretreatment of the biomasses from each part (leaves, stems, and roots) of wild-type and transgenic tobacco plants was conducted by using 3 mL 1% H_2_SO_4/_g biomass (*v*/*w*) and shaking for 3 h at room temperature.

Enzymatic hydrolysis was performed in a 10.0 mL total volume containing 1% (*w*/*v*) dry matter, with or without cellulase (Celluclast, Novozymes A/S, Bagsvaerd, Denmark) (17.74 mg/g biomass), in 0.05 M citrate phosphate buffer (pH 4.8) at 37 °C, with shaking at 200 rpm for 24 h in a 50 mL conical tube. The enzymatic hydrolysis yields were measured via high-performance liquid chromatography (HPLC) by refractive index detector (2414; Water, Milford, MA, USA), REZEX RPM (Phenomenex, Torrance, CA, USA) column (300 × 7.8 mm) with program of 85 °C and flow rate of 0.6 mL/min.

### 2.6. Techno-Economic Assessment—Costs for Planting and Biomass Yields

Production of biomass was designed for two scenarios: a small-scale scenario in which 100 plants were cultivated in a batch/season in greenhouse and an assumed large-scale scenario with 30,000 plants in a batch/season in a 1-ha of field. For the small-scale scenario, seedlings at 14 DAG from the growth chamber were transferred to greenhouse under the conditions mentioned above, and the costs of electricity and water were counted from this time. The prices and costs of electricity and water were determined based on the prices for January–December 2017 in Chonnam National University, Bukgu, Gwangju, Republic of Korea, from January–December 2017. Due to the self-production, the price of seeds (wild-type and transgenic tobacco plants) was not counted. The prices of soil, spots, and fertilizer were listed. The numbers of days from germination to harvest, at the time when fruits and leaves are entering senescence, is very important for influencing to the costs of electricity and water usage. In the small-scale scenario, the cost for labor was not counted. In the large-scale scenario, the cost for field was counted, as were other costs for utilities (water, electricity, and fertilizer), but the cost for labor made up the largest portion, with at least 2 persons providing labor during one batch cultivation, and based on basic salary for 2017 (~US$ 6.25/h, US$ 50/8h/working day/person) in the Republic of Korea. At harvest, biomass yields were measured in fresh/dry weight (FW/DW, kg).

## 3. Results and Discussion

### 3.1. Vector Construction for the Overexpression of Thermostable β-Glucosidase T. maritima BglB Targeted to the Chloroplasts and Vacuoles in Tobacco Plants and β-Glucosidase Enzymatic Activity

**Figure 1:**
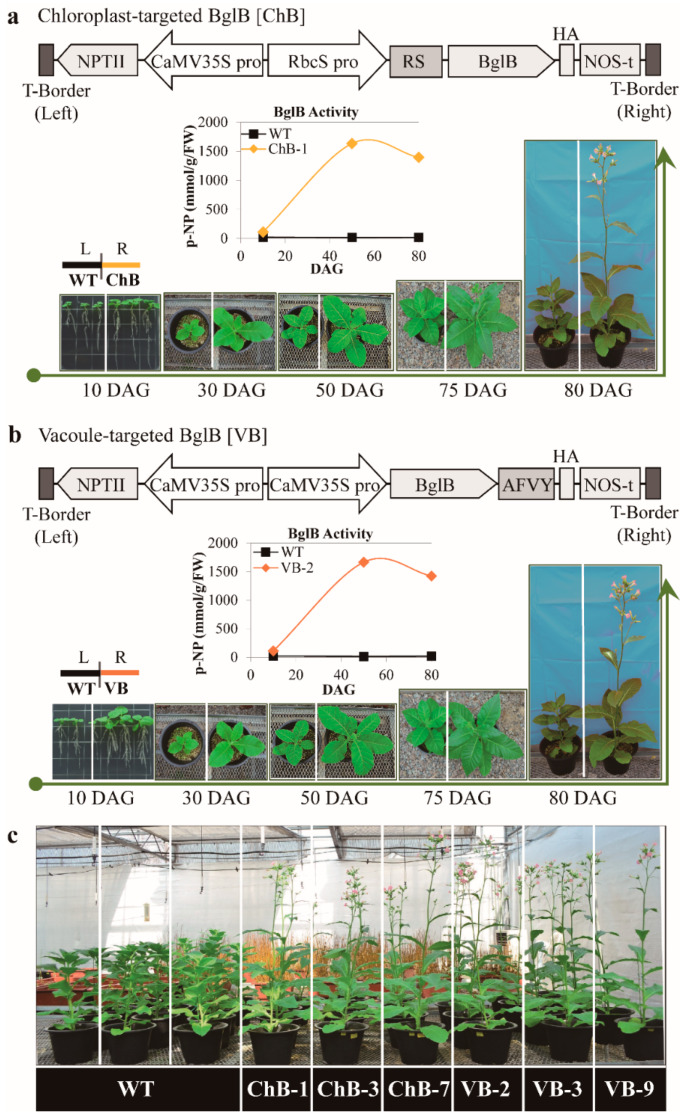
Vector construction, and β-glucosidase activity in, and growth and development of transgenic and wild-type (WT) tobacco plants. (**a**) Vector construction, and β-glucosidase activity in, and growth and development of transgenic tobacco plants overexpressing *T. maritima BglB* targeted to the chloroplast in the T1 generation at 10–80 days after germination (DAG) in comparison with those of wild-type tobacco plants, under greenhouse conditions. (**b**) Vector construction and β-glucosidase activity in, and growth and development of transgenic tobacco plants overexpressing *T. maritima BglB* targeted to the vacuoles in the T1 generation at 10–80 DAG, in comparison with those of wild-type tobacco plants, under greenhouse conditions. (**c**) Growth and development of the wild-type and T2 to T4 generations of transgenic tobacco plants overexpressing *T. maritima BglB* targeted to the chloroplasts and vacuoles, under greenhouse conditions. (ChB: Chloroplast-targeted *BglB*, VB: Vacoule-targeted *BglB*, T1: first generation progeny plants).

To transform the *T. maritima BglB* gene and target it to the chloroplasts (ChB) and vacuoles (VB) of tobacco plants, the following two vectors containing polypeptides were used: (1) the n-terminal transit peptide isolated from a small subunit of the Rubisco complex, RS: MASSMLSSATMVASPAQATMVAPFNGLKSSAAFPATRKANNDITSITSNGGRVNCMQVWPPIGKKKGETLSYLPDLTDSE, and (2) the C-terminal polypeptide, AFVY; these vectors target the desired gene to the chloroplasts and vacuoles, respectively. The constructed vectors, adapted according to previous studies [7,21], are shown in Figure 1a,b. Successful targeting of *BglB* to these organelles was confirmed [7,21,24]. The results indicated that the transgenic plants not only produced *BglB* for enzymatic processing but also changed the physical growth and development of the transgenic tobacco plants.

The enzymatic activity of *BglB* was examined during the growth of the wild-type and transgenic plants, and the results are presented in Figure 1a,b. Interestingly, the highest activity of *BglB* was recorded approximately 40–60 days after germination (DAG) and before the transgenic plants produced buds and flowers, in both chloroplast- and vacuole-targeted tobacco plants. This indicated that the heterologous overexpression of *BglB* might trigger metabolic changes that result in an enhanced development of transgenic plants. It is important to note that the enzymatic activity of *BglB* and the pronounced changes in plant phenotypes were maintained for many generations of the transgenic plants, indicating that the genotypes of the transgenic plants and the effects of heterologous *BglB* were stable. This is important for developing applications of transgenic plants following genetic transformation.

### 3.2. Phenotypes of Transgenic Plants Overexpressing T. maritima BglB Targeted to the Chloroplasts and Vacuoles Enhanced Growth and Biomass Yield

After the successful transformation and targeting of *BglB* to the chloroplasts and vacuoles, several generations (T1 to T4) of the transgenic tobacco, and wild-type tobacco plants were grown under standard conditions in a greenhouse. The transgenic plants showed pronounced phenotypic changes compared with those of the wild-type tobacco plants. The phenotypes including the height of stem; weight of leaves, stem, roots, fruits, and total biomass; number of fruits; area of leaves; and time of flowering of at least three transgenic lines (30 transgenic plants from each line) and wild-type tobacco plants were analyzed for four generations (T1 to T4). Overall, the growth and development of transgenic tobacco plants in the T1 generation were significantly enhanced compared with those of the wild-type tobacco plants (Figure 1a,b). The transgenic plants had larger leaves and taller stems at all sampling time points, and they also showed earlier budding and flowering; these changes were observed very early during growth. Even at 10 DAG, transgenic seedlings had larger leaves, longer roots, and more lateral roots. Figure 1c depicts the three subsequent generations (T2 to T4) of each transgenic line, which showed enhanced development (larger plants and earlier flowering) in comparison with that of the wild-type tobacco plants. It is also noteworthy that the enhanced growth and development of the transgenic plants was supported by their larger root systems, as indicated by the synchronous development of the transgenic plants (Figure S1).

**Figure 2:**
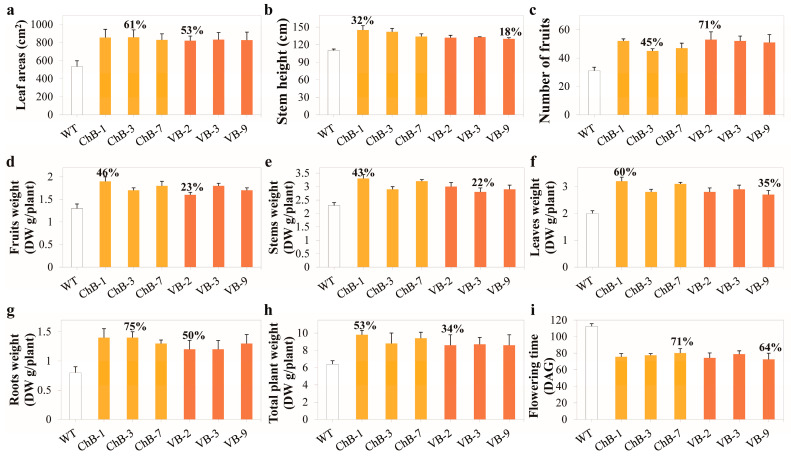
Phenotypic changes in the transgenic tobacco plants overexpressing *T. maritima BglB* targeted to the chloroplasts and vacuoles in comparison with those in the wild-type tobacco plants. (**a**) leaf areas; (**b**) stem height; (**c**) fruits numbers; (**d**) fruits weight; (**e**) stem weight; (**f**) leaves weight; (**g**) roots weight; (**h**) total plant weight; and (**i**) flowering time. Average values were calculated in triplicate (*n* = 3) for each transgenic line and wild-type tobacco plants. The bars of each column show standard deviation (SD).

Detailed results of the pronounced phenotypic changes in the transgenic tobacco plants in the T1 generation in comparison with the wild-type tobacco plants are presented in Figure 2. Leaf area was measured at flowering, and the results indicated a significant increase in leaf area in the transgenic tobacco plants as compared with the wild-type plants. The transgenic tobacco plants had leaves that were 53% (VB-2 line) to 61% (ChB-4 line) larger than those of the wild-type plants (Figure 2a). At harvest, the stem height of the mature *BglB* transgenic lines was 18% (VB-9 line) to 32% (ChB-1 line) higher than that of the wild-type plants (Figure 2b). The fruit number and biomass dry weight (DW) were also measured at harvest. All parts (fruits, leaves, stems, and roots) were separated, dried using freeze dryers, and analyzed separately (although differences in fresh weights were pronounced, these data are not shown because biomass samples might have a different moisture content). The number of fruits was 45% (ChB-4 line) to 71% (VB-2 line) higher in the transgenic plants than in the wild-type plants (Figure 2c), and the DW of tobacco fruits, which might have included seeds, was 23% (VB-2 line) to 46% (ChB-1 line) higher in the transgenic plants than in the wild-type plants (Figure 2d). The DW of stems, leaves, and roots of the transgenic plants was 22% (VB-3 line) to 43% (ChB-1 line), 35% (VB-9 line) to 60% (ChB-1 line), and 50% (VB-2 line) to 75% (ChB-4 line) higher than that of the wild-type tobacco plants, respectively (Figure 2e–g). Consequently, the total DW of transgenic tobacco plants at harvest was 34% (VB-9 line) to 53% (ChB-1 line) higher than that of the wild-type tobacco plants (Figure 2h). The same phenomenon of enhanced plant growth and development, accompanied by higher reproductive rates, was also observed in the subsequent generations (T2 to T4) of the transgenic plants (Figure S2).

Similarly, by reducing photosynthesis inefficiency, South et al. [8] reported an increase in biomass production by 40% in transgenic tobacco compared with that in wild-type tobacco plants, probably owing to an increase in the photosynthesis rate and plant size. Recently, the use of genetic engineering techniques to reduce the expression of just one pectin biosynthesis gene, *galacturonosyltransferase 4* (*GAUT4*), in switchgrass and poplar led to an increase in biomass yields. That is, the dry biomass yield of the transgenic plants was, on an average, 59% higher than that of wild-type switchgrass and poplar [25]. Nevertheless, from a technical point of view, our approach seems to be considerably simple and achieves multiple objectives by increasing plant biomass and β-glucosidase production.

Here, we observed an interesting trait in the transgenic tobacco plants (in both chloroplast and vacuole targeted lines), that is, they flowered earlier than the wild-type tobacco plants. After germination, the flowering time was 29% (ChB-6 line) to 36% (VB-9 line) shorter in the transgenic tobacco plants than in the wild-type tobacco plants (Figure 2i). The flowering stage is an important development stage in the life cycle of plants; it is the stage at which plants progress to the reproductive stage after reaching maturity [5]. Thus, the trait of earlier flowering, together with other enhanced growth traits described above, is highly significant. Under the same cultivation conditions, transgenic tobacco plants presented a shorter life cycle, but higher growth rates and better improvement, including higher biomass yield. Therefore, the transgenic plants may have superior traits in comparison with the wild-type tobacco plants, and similar positive effects might also be achieved in other crops. Moreover, plants often produce flowers under unfavorable or stressful conditions (e.g., drought and heat stresses), whereas our transgenic tobacco plants showed good growth and development under normal conditions. This indicates that the existence of heterologous *BglB* in the chloroplasts and vacuoles could trigger an unclear synchronous mechanism (or a series of mechanisms) that enhances growth and development in the transgenic tobacco plants.

### 3.3. Involvement of HMG, F-Box, and MDH in the Transgenic Plants

**Figure 3:**
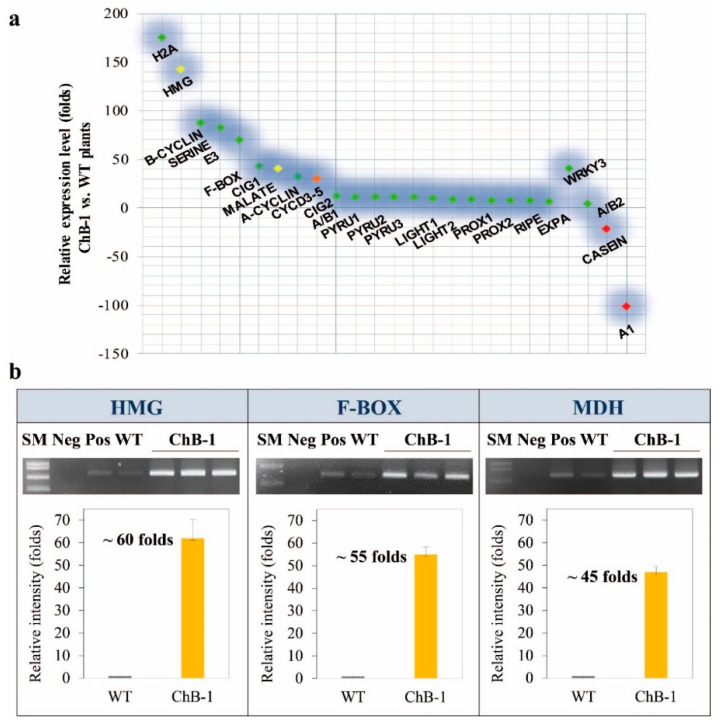
Results of mRNA microarray and mRNA expression levels. (**a**) A summary of the results of mRNA microarray conducted for the relative expression level of genes in the transgenic tobacco plants overexpressing *T. maritima BglB* targeted to the chloroplasts in comparison with that in the wild-type tobacco plants, indicating that a series of genes was up-regulated, especially high-mobility group (HMG), F-Box, and malate dehydrogenase (MDH). (**b**) The results of mRNA expression levels of HMG, F-Box, and MDH in the transgenic tobacco plants overexpressing *T. maritima BglB* targeted to the chloroplasts in comparison with those in the wild-type tobacco plants; this assay was conducted by extracting mRNA, generating a cDNA library, and running RT-PCR.

We evaluated whether there was any change in genomic/protein expression levels in plant cells with abundant heterologous *BglB* (especially in the chloroplasts and vacuoles) and identified molecular mechanisms that trigger enhanced growth and development in the transgenic plants. Figure 3a shows the relative expression levels of 25 genes, which were selected from hundreds of genes recorded by mRNA microchip experiments. These 25 genes were either upregulated- or downregulated in the transgenic tobacco plants of the ChB-1 line in comparison with those in the wild-type plants, confirming that there were some changes in the transgenic tobacco plants. It is possible that almost all these upregulated and downregulated genes are controlled by changes in the level of homeostasis hormone, but the exact mechanisms remain unclear. Among these genes, three up-regulated genes, high-mobility group 1 (*HMG-1*), acetyltransferase F-Box, and malate dehydrogenase (*MDH*), which are tightly controlled by the elevation in hormone levels, should be considered for future research. The expression of these genes was significantly higher (45–60 times) in the transgenic ChB-1 line than in the wild-type tobacco plants (Figure 3b), as evidenced by the results of the mRNA micro-array analysis. Similar results were also obtained from the vacuole targeted transgenic lines (Figure S3).

**Figure 4:**
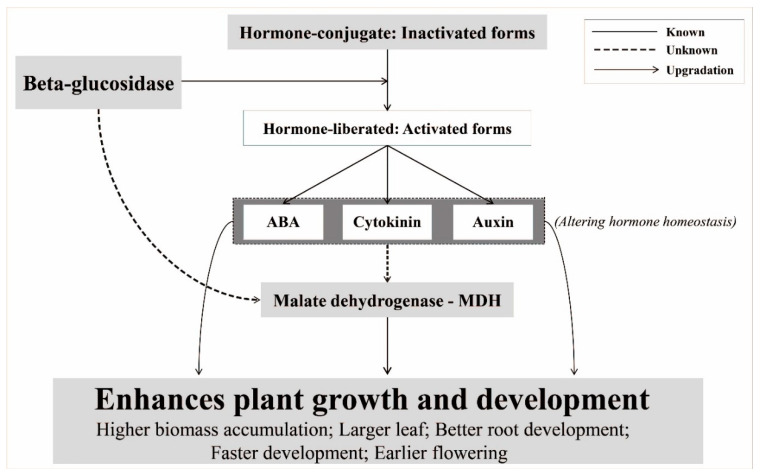
Hypothesis of the mechanism by which overproduced β-glucosidase enhances growth and development in transgenic plants.

*HMG* and F-box belong to a large group of genes, which include many genes that are normally upregulated by hormones during plant growth and development, but the effects of these genes on the superior growth of transgenic plants remain unclear [26,27,28]. Several studies have provided evidence of the functional roles of MDH in the increase/decrease in biomass yields in plants [14,29]. MDH (E.C. 1.1.1.37) catalyzes the interconversion of malate and oxaloacetate (OAA), coupled with reduction/oxidation of the NAD pool, and it is involved in two major processes in plants, namely, oxidizing malate from the fumarase reaction to OAA to ultimately form citrate and reducing OAA to malate to supply NAD^+^ for Gly decarboxylase [30]. Previous studies have reported that the knockdown of *MDH* in many plants (Arabidopsis, cotton, tomato, alfalfa, and tobacco) led to reduced biomass weight and root length [14,15], whereas the overexpression of *MDH* significantly increased biomass weight and enhanced growth (Table S1) [16,27,31]. Moreover, a direct influence of β-glucosidase on MDH has never been confirmed although an abundant expression of heterologous *BglB* and *MDH* was observed in this study. Based on these observations, we propose a hypothesis to explain how the overexpression of heterologous *BglB* could enhance the growth and development of the transgenic tobacco plants, via the following two main mechanisms. 1) An abundance of heterologous β-glucosidase *BglB* hydrolyzes hormone-conjugates (inactivated forms) to release hormones (activated forms) that function as factors or lead to up-regulated MDH expression levels, enhancing plant growth and development, and 2) an abundance of heterologous β-glucosidase *BglB* serves as a factor upregulating *MDH*, which in turn enhances plants growth and development. This hypothesis, illustrated in Figure 4, provides a novel explanation for the enhanced growth and development of the transgenic tobacco plants by heterologous overexpression of β-glucosidase, which has already been implicated in key developmental processes in plants.

### 3.4. Enhanced Saccharification in the Enzymatic Conversion of Biomass from Transgenic Plants Expressing β-Glucosidase T. maritima BglB

Our previous studies confirmed that *BglB* maintained its function in vitro by hydrolyzing p-NP [7,21], which indicates that the heterologous β-glucosidase *BglB* may auto-hydrolyze or it could be used to hydrolyze plant biomass. To examine the hydrolysis conversion efficiency of biomass from wild-type and transgenic tobacco plants, we used mild acid pretreatment and saccharification (with the same amount of additional cellulase). The leaves, stems, and roots from different stages (30 DAG, flowering, and harvest) of the wild-type and transgenic lines (ChB-1 and VB-2 lines) were collected and freeze-dried for further experiments. First, the chemical compositions of wild-type and transgenic tobacco plants was investigated. As shown in Table 1, among the wild-type and transgenic lines, a higher xylose content was observed in the stems and roots than in the leaves, making the total carbohydrate composition slightly higher in the stems and roots than in the leaves. Moreover, the total carbohydrate content was slightly higher in the transgenic lines than in the wild-type plants. No significant differences were observed in lignin, extractive compounds, or ash between the wild-type and transgenic tobacco plants (data not shown). Before conducting saccharification, the total protein extracts obtained from un-pretreated and pretreated biomass of the wild-type and transgenic tobacco plants were used to check the enzymatic activity of *BglB*. The results showed superior enzymatic activity in the biomass from the transgenic plants than from the wild-type plants. A higher activity of *BglB* was also observed in the leaves, stems, and roots of the transgenic tobacco plants until harvest, indicating that the heterologous *BglB* retains its functions during plant growth and development.

**Figure 5:**
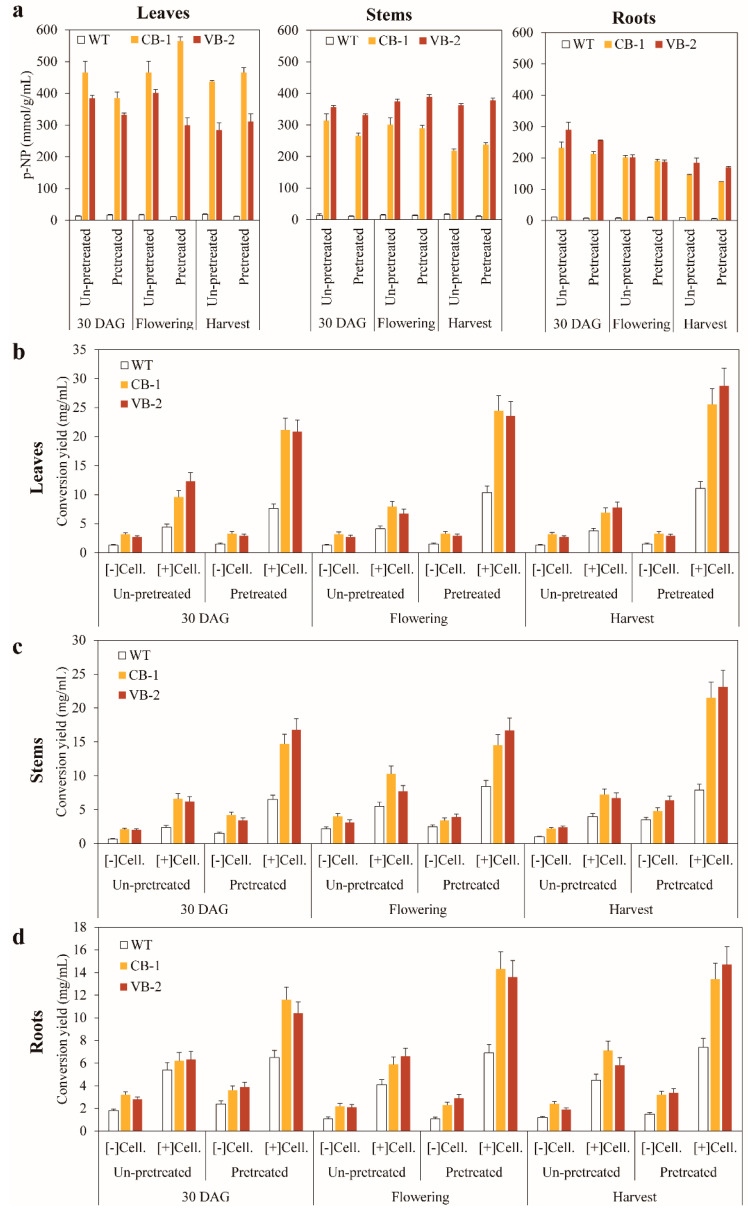
(**a**) β-Glucosidase activity in samples of leaves, stem, and roots of the wild-type and transgenic tobacco plants overexpressing *T. maritima BglB* targeted to the chloroplasts and vacuoles. Comparison of conversion yield of (**b**) leaves, (**c**) stems, and (**d**) roots after saccharification with and without additional cellulase in un-pretreated and pretreated biomasses from the wild-type and transgenic tobacco plants overexpressing *T. maritima BglB* targeted to the chloroplasts and vacuoles. Average values were calculated in triplicate (*n* = 3) for each transgenic line and wild-type tobacco plants.

These results are significant, because the stability and proper function retention of heterologous cellulolytic enzymes are important during plant growth and development, with the optimal activities of the enzymes remaining until harvest. In addition, *BglB* from the leaves might present higher enzymatic activities than that from the stems and roots of the plants. Moreover, this was accompanied by slight changes (decrease or increase) in the activity of *BglB* from the un-pretreated and pretreated biomass, indicating that the heterologous *BglB* still retained its functions after freeze drying and pretreatment (Figure 5a).

Next, the wild-type and transgenic tobacco plants were used as a biomass source for saccharification. All the biomass samples were hydrolyzed using the same amount of additional cellulase (and without additional cellulase as controls) and saccharification conditions. The results for conversion yield are presented in Figure 5b–d. Generally, the conversion yields in each part was significantly higher in the transgenic plants samples than in the wild-type tobacco plant samples, in both un-pretreated and pretreated samples, and with or without cellulase. Additionally, there was no significant difference in the conversion yield between the chloroplast- and vacuole-targeted plant samples, indicating that it is feasible to use biomass samples from both transgenic tobacco plants. With additional cellulase, the conversion yield was significantly enhanced in both un-pretreated and pretreated biomass samples. The pretreatment process also improved the conversion yield in all plant samples. A comparison of biomass sampling points of the transgenic tobacco plants revealed that the conversion yield at harvest was higher than that at 30 DAG and flowering. The highest conversion yields were 28.7, 23.1, and 14.7 mg/mL in the leaves, stems, and roots, respectively, at harvest. Furthermore, the leaves presented higher conversion yield than the stems and roots for biomass samples. The results of saccharification clearly indicated the superior efficiency of biomass from the transgenic tobacco plants as a raw material for saccharification. The synergistic effects may occur depending on the presence of abundant heterologous *BglB* within plant cells and the additional cellulase. This is a novel finding as the saccharification results were previously based on fresh biomass samples [18].

The considerably higher conversion yield after saccharification in the biomass samples from the transgenic tobacco plants requires an explanation. Several previous studies have reported a higher conversion in the β-glucosidases transgenic plant and provided evidence that the morphology of the transgenic biomass was altered by the influence of β-glucosidases during plant growth and development [5,18,32]. Our results indicated no significant difference between carbohydrate content of biomass samples from wild-type and transgenic tobacco plants at the three sampling points (Table 1), suggesting that other unknown causes might be involved in the enhancement of saccharification. Distinctively, altered plant cell wall polymers, represented by the degree of polymerization (DP), could provide an explanation. With significantly lower DP, higher conversion rates were observed in transgenic than in wild-type rice straw [18]. By down-regulating pectin biosynthesis via *GAUT4*, Biswal et al. reported a reduction in the synthesis of homogalacturonan (HG) and rhamnogalacturonan II (RGII), which led to a decrease in recalcitrance in plant cell wall biomass, consequently increasing saccharification [25].

### 3.5. Difference in the Fourier Transformed Infrared (FT-IR) Spectra between the Transgenic and Wild-Type Tobacco Plants

**Figure 6:**
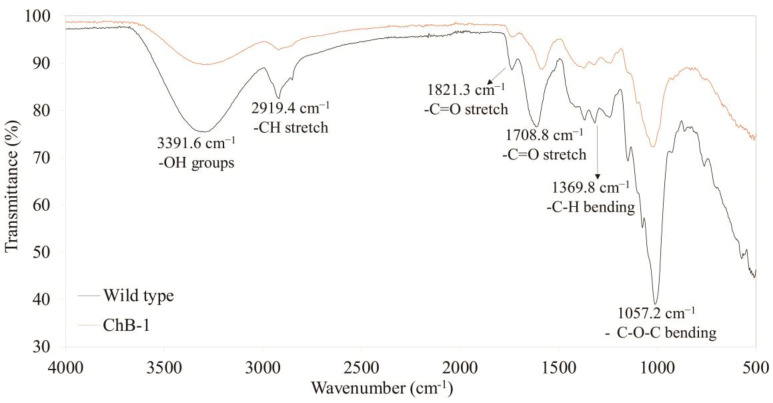
Fourier-transform infrared (FT-IR) spectra of biomass obtained from the wild-type and transgenic tobacco plants overexpressing *T. maritima BglB* targeted to the chloroplasts (ChB-1 line). Wild-type: black line, transgenic: red line.

Figure 6 distinguishes between biomass from the wild-type and transgenic tobacco plants, with several apparent differences in their FT-IR spectra, including the disappearance of or significant reduction in peaks representing lignin and other non-saccharide compounds [2]. Adsorption peaks around 3,241.3–3,391.6 cm^−1^ were clearly observed in the wild-type tobacco plants, but they were reduced in the ChB-1 tobacco plants, indicating a lower abundance of free and intermolecular bonded O-H groups in the ChB-1 plants than in the wild-type plants. Another remarkable observation was a peak at 2919.4 cm^−1^, assigned to the stretching vibration of the lignin C-H group, and it was prominent in the wild-type plant but almost absent in the ChB-1 tobacco plants. This indicates that the lignin content was significantly lower in the ChB-1 than in the wild-type tobacco plants. Moreover, the same trends were observed for peaks at around 1821.3 and 1708.8 cm^−1^ (corresponding to lignin C=O stretching), 1369.8 cm^−1^ (corresponding to the lignin C-H bending), and 1057.2 cm^−1^ (corresponding to the lignin C-O-C bending), accompanied with differences in the pattern of peaks from 831.2–380.0 cm^−1^, and they were assigned to the bending modes of aromatic compounds. From these results obtained using biomass samples from the heterologous *BglB* transgenic tobacco plant, especially samples pretreated with mild acid, it can be inferred that saccharification was considerably enhanced. The probable reasons for this enhancement are as followed: (1) changes in chemical components that make up biomass structures, and (2) the synergistic effect of additional cellulase and heterologous *BglB* within plant cells.

### 3.6. Techno–Economic Assessment of the Increase in Biomass Production in the Transgenic Tobacco Plants as Compared with the Wild-Type Tobacco Plants

**Figure 7:**
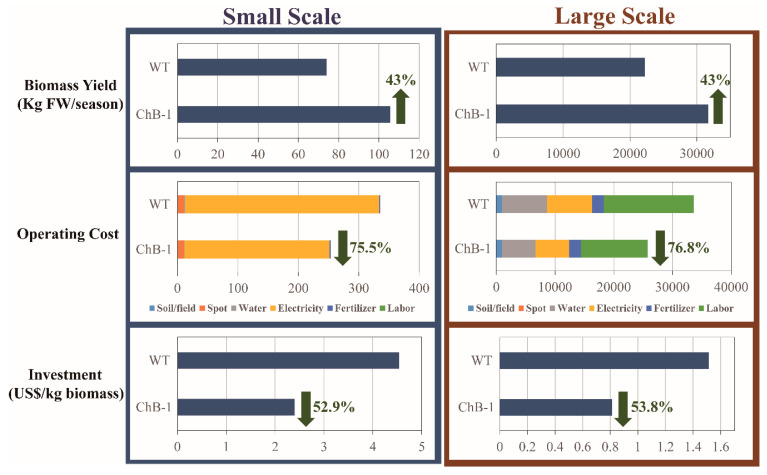
Comparison of the results of techno–economic assessment of investment costs of planting and biomass yield between the wild-type and transgenic tobacco plants overexpressing *T. maritima BglB* targeted to the chloroplasts (ChB-1 line).

For the production of value-added products in new manufacturing facilities/systems, techno–economic assessments are critical to establish the requirements, constraints, and costs involved in production. Enhanced biomass accumulation and heterologous *BglB* activity were found in the transgenic tobacco plants; therefore, we conducted a techno–economic assessment for the costs of planting and biomass yield. The process design and cost analysis were evaluated for two scenarios: a small-scale (cultivation of 100 plants in a batch/season in a greenhouse) and theoretical large-scale (cultivation of 30,000 plants in a batch/season on a 1-ha field) (Figure 7).

In the small–scale scenario, the total biomass yield for one batch/season was approximately 74.0 and 105.7 kg fresh weight (FW) for the wild-type and transgenic ChB-1 line, respectively. The same trend was observed for the DW, with approximately 4.4 and 6.9 kg dry mass harvested from the wild-type and transgenic tobacco plants, respectively. Moreover, because of the shorter time from germination to harvest of the transgenic tobacco plants, three cultivation seasons can be achieved when using these transgenic tobacco plants in comparison with only two seasons with wild-type tobacco plants (approximately 114 and 153 DAG for the transgenic and wild-type tobacco plants, respectively). Thus, approximately 148.0 (two seasons) and 317.2 kg FW (three seasons) per year, can be obtained from the wild-type and transgenic tobacco plants, respectively, which is equivalent to an increase of 2.14 fold.

Next, we calculated the total operating costs for one batch/season. The input costs included seeds, utilities (soil, spot, water, electricity, and fertilizer), labor, and number of days/life cycle. The cost of seeds and labor was not included in the analysis of the small-scale scenario. The total operating costs for one batch/season was US$ 335.8 and US$ 253.5 for the wild-type and transgenic tobacco plants, respectively. The cost of electricity made up the highest proportion for both wild-type and transgenic tobacco plants (95.7% and 94.4%, respectively). In addition, the electricity cost for the transgenic plants was lower than that for the wild-type plants, as the time required for maturation/harvest was shorter for the transgenic plants. These results indicated that the operating cost was significantly lower (by approximately 75.5%) when cultivating the transgenic tobacco plants than the wild-type tobacco plants. If the total operating costs are compared over a year, transgenic tobacco plants (US $760.5 for three seasons) are more expensive than wild-type plants (US $671.6 for two seasons). However, when comparing the investment costs for the production of 1 kg FW, the values were US$ 4.54 and US$ 2.40 for the wild-type and transgenic tobacco plants, respectively. Thus, there was a considerably lower investment cost when cultivating the transgenic tobacco plants, which required only approximately 52.9% of the investment for the wild-type tobacco plants.

The theoretical large-scale scenario showed a trend similar to that of the small-scale scenario. The biomass yield tended to be the same as described for the small-scale scenario. With respect to operating costs, there was a change compared with that of the small–scale scenario. The ratio of electricity cost was reduced, and the ratio of water and fertilizer costs was increased. In addition, the cost of labor was the main expense included in the total investment (representing 45.5 and 44.2% of the total investment for cultivation of the wild-type and transgenic plants, respectively). However, the ratio of total operating costs between the wild-type and transgenic plants was similar to that of the small-scale scenario. It led to significantly lower total operating costs (76.8%) when cultivating the transgenic tobacco plants than the wild-type tobacco plants. Furthermore, the investment costs for 1 kg FW biomass production were considerably reduced in a comparison between the small-scale and large-scale scenario: from US$ 4.50 to US$ 1.50 for the wild-type tobacco plants, respectively, and US$ 2.40 to US$ 0.80 for transgenic tobacco plants ChB-1 line, respectively. These achievements suggest good prospects for the applications of *T. maritima BglB* transgenic tobacco plants and indicate that the method used in this study could be applied to enhance biomass production and heterologous *T. maritima BglB* expression not only in tobacco plants but also in many other plants.

## 4. Conclusions

In summary, enhanced biomass production and saccharification in the transgenic tobacco plants overexpressing *T. maritima BglB* targeted to the chloroplasts and vacuoles clearly indicate that this approach has a great potential and that transformed thermostable β-glucosidase can trigger significant changes in phenotypic characteristics. The significant increases in biomass production in the transgenic tobacco plants was found to be due to substantial changes in the genome transcription levels owing to the abundance of heterologous β-glucosidase within plant cells. Evidence of the reduction in operating costs for plant biomass production, and enhancement of saccharification in β-glucosidase-producing plant biomass, suggests that our approach could be applied for multiple purposes in biorefinery processes. This approach could also be applied to many other plant species to increase biomass yield and overproduce β-glucosidase *BglB* for lignocellulosic conversion.

## Figures and Tables

**Table 1:** Carbohydrate content of plant organs obtained from wild-type and transgenic tobacco plants overexpressing *T. maritima BglB* targeting the chloroplasts and vacuoles.

Heading	%	WT	ChB-1	ChB-3	ChB-7	VB-2	VB-3	VB-9
Leaves	Rhamnose	2.2 ± 0.2	2.3 ± 0.3	2.2 ± 0.2	2.7 ± 0.3	2.5 ± 0.3	2.5 ± 0.4	2.0 ± 0.0
	Arabinose	1.3 ± 0.0	1.5 ± 0.1	1.3 ± 0.1	1.6 ± 0.2	1.0 ± 0.0	1.1 ± 0.2	1.3 ± 0.1
	Xylose	1.5 ± 0.2	1.6 ± 0.1	1.5 ± 0.1	1.5 ± 0.2	1.4 ± 0.1	1.5 ± 0.1	1.2 ± 0.1
	Mannose	0.6 ± 0.0	1.1 ± 0.2	1.1 ± 0.1	0.6 ± 0.0	0.6 ± 0.0	0.4 ± 0.0	1.0 ± 0.0
	Galactose	2.2 ± 0.0	2.0 ± 0.0	2.0 ± 0.0	2.1 ± 0.1	2.1 ± 0.0	2.0 ± 0.0	2.0 ± 0.1
	Glucose	46.3 ± 1.1	46.0 ± 2.1	45.1 ± 2.0	45.1 ± 1.7	45.8 ± 5.1	45.1 ± 2.5	45.3 ± 1.1
	Total	54.1 ± 1.3	54.5 ± 3.1	53.2 ± 3.0	53.6 ± 2.1	53.4 ± 4.8	52.6 ± 3.3	52.8 ± 1.5
Stems	Rhamnose	1.9 ± 0.1	1.3 ± 0.2	1.0 ± 0.1	1.3 ± 0.1	1.2 ± 0.0	1.1 ± 0.1	1.3 ± 0.2
	Arabinose	0.8 ± 0.0	0.9 ± 0.0	1.1 ± 0.0	1.1 ± 0.0	1.3 ± 0.1	0.7 ± 0.1	1.2 ± 0.0
	Xylose	5.4 ± 0.5	5.5 ± 0.4	6.3 ± 0.2	7.2 ± 1.4	6.4 ± 0.7	5.4 ± 0.2	6.0 ± 0.2
	Mannose	1.1 ± 0.2	1.4 ± 0.1	1.3 ± 0.0	1.3 ± 0.2	1.0 ± 0.1	1.3 ± 0.0	1.3 ± 0.1
	Galactose	1.3 ± 0.4	1.5 ± 0.2	1.2 ± 0.1	1.3 ± 0.0	1.2 ± 0.1	1.2 ± 0.0	1.2 ± 0.1
	Glucose	45.1 ± 2.7	45.3 ± 1.3	45.8 ± 2.1	46.2 ± 1.4	45.8 ± 1.7	46.1 ± 2.9	44.1 ± 1.8
	Total	55.6 ± 3.1	55.9 ± 2.1	56.7 ± 3.5	58.4 ± 2.4	56.9 ± 3.7	55.8 ± 3.4	55.1 ± 2.6
Roots	Rhamnose	1.2 ± 0.0	1.2 ± 0.1	1.5 ± 0.0	1.2 ± 0.0	1.5 ± 0.2	1.5 ± 0.3	1.1 ± 0.1
	Arabinose	0.9 ± 0.0	1.1 ± 0.1	1.0 ± 0.0	1.1 ± 0.0	1.2 ± 0.2	0.9 ± 0.1	0.7 ± 0.2
	Xylose	6.5 ± 1.3	7.0 ± 0.2	6.8 ± 0.5	6.5 ± 0.8	6.4 ± 0.8	7.1 ± 0.2	7.1 ± 0.3
	Mannose	1.0 ± 0.1	1.2 ± 0.1	1.3 ± 0.0	1.4 ± 0.1	0.8 ± 0.0	1.3 ± 0.1	1.2 ± 0.1
	Galactose	1.3 ± 0.1	1.0 ± 0.0	1.2 ± 0.1	1.2 ± 0.1	0.7 ± 0.0	0.8 ± 0.0	0.9 ± 0.0
	Glucose	45.7 ± 2.8	46.0 ± 3.9	45.3 ± 2.5	45.1 ± 0.9	44.9 ± 0.7	45.8 ± 2.4	45.9 ± 4.1
	Total	56.6 ± 3.8	57.5±4.1	57.1±3.3	56.5±1.6	55.5±3.8	57.4±4.1	57.2±4.8

## Supplementary Materials

The following are available online at https://www.mdpi.com/2218-273X/10/5/806/s1, Root development in the wild-type and T1 generation of transgenic tobacco plants overexpressing *T. maritima BglB* targeted to the chloroplasts and vacuoles after harvest, β-Glucosidase enzymatic activities and phenotype characteristics of the wild-type and transgenic tobacco plants overexpressing *T. maritima BglB* targeted to the chloroplasts and vacuoles (T2 to T4 generations), Results of mRNA expression levels of HMG, F-Box, and MDH in the transgenic tobacco plants overexpressing *T. maritima BglB* targeted to the vacuole in comparison with those in the wild-type tobacco plants, Functional role of MDH (EC 1.1.1.37).
