# Supplementary material for: Enhanced Biomass Yield of and Saccharification in Transgenic Tobacco Over-Expressing β-Glucosidase
Source: Biomolecules. 2020 May 23;10(5):806. doi: 10.3390/biom10050806 (PMC7278181; doi:10.3390/biom10050806)
Supplement: Supplementary file 1 [file biomolecules-10-00806-s001.pdf]

Supplementary Table S1: Functional role of MDH (EC 1.1.1.37).

Functional roles of MDH (EC 1.1.1.37)			
	Plants	Characteristic	References
Knock-down	Cotton	Reduce 30% fresh biomass weight	[1]
	Arabidopsis	Reduce 50% biomass weight	[2]
	Tomato	Reduce: 50% root length; 50% root dry weight	[3]
Overexpression	Cotton	Increase 20% fresh biomass weight	[1]
	Alfalfa	Increase 120% dry biomass weight; 100% roots dry weight	[4]
	Arabidopsis	Increase 70% dry biomass weight	[5]
	Tobacco	Increase 20% fresh biomass weight; 50% root growth rate	[6]

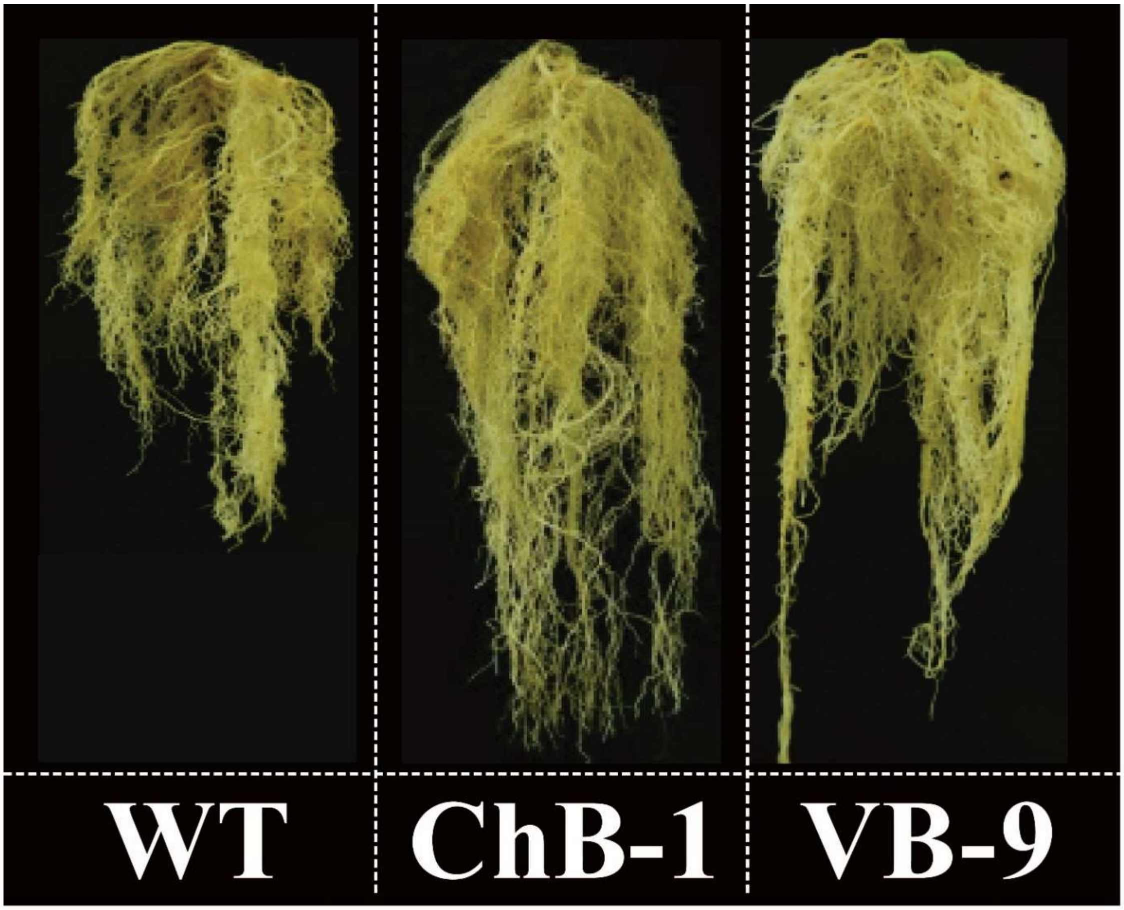

Supplemental Figure S1. Root development in the wild-type and T1 generation of transgenic tobacco plants overexpressing *T. maritima* BglB targeted to the chloroplasts and vacuoles after harvest.

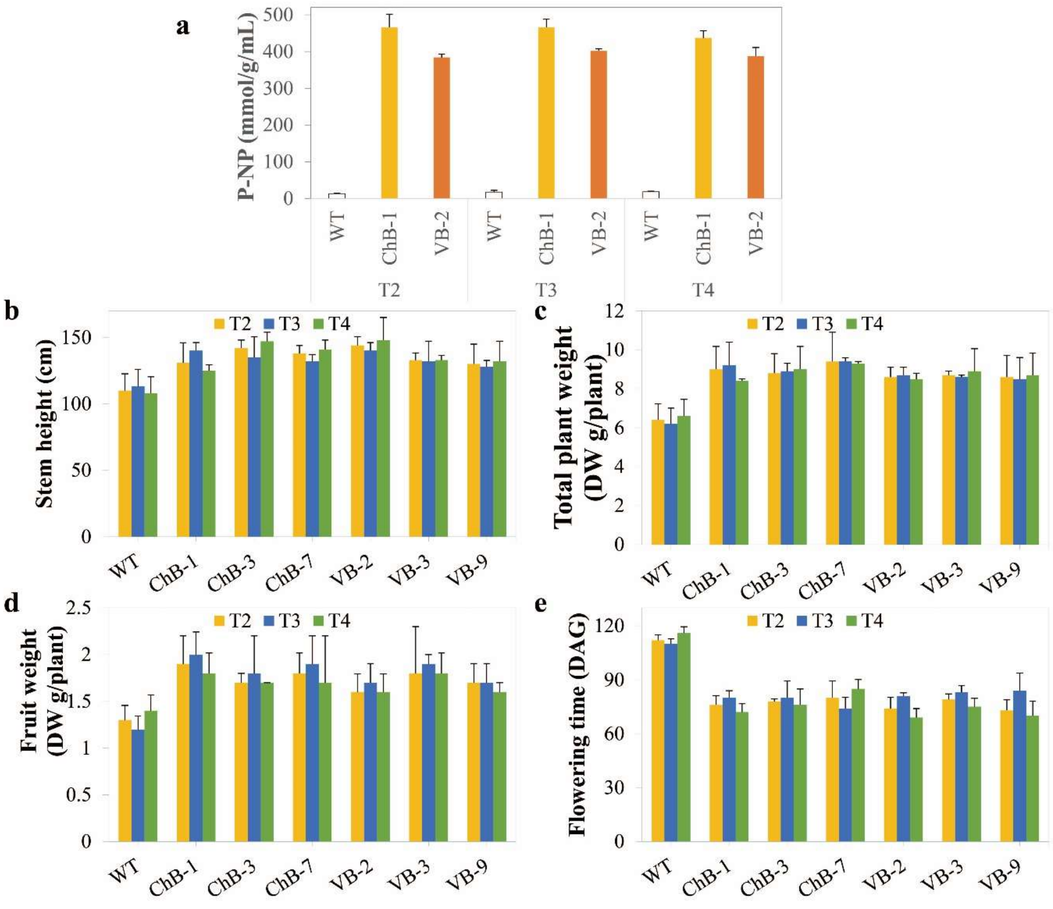

Supplemental Figure S2. (a) β-Glucosidase enzymatic activities and (b-e) phenotype characteristics of the wild-type and transgenic tobacco plants overexpressing *T. maritima* BglB targeted to the chloroplasts and vacuoles (T2 to T4 generations).

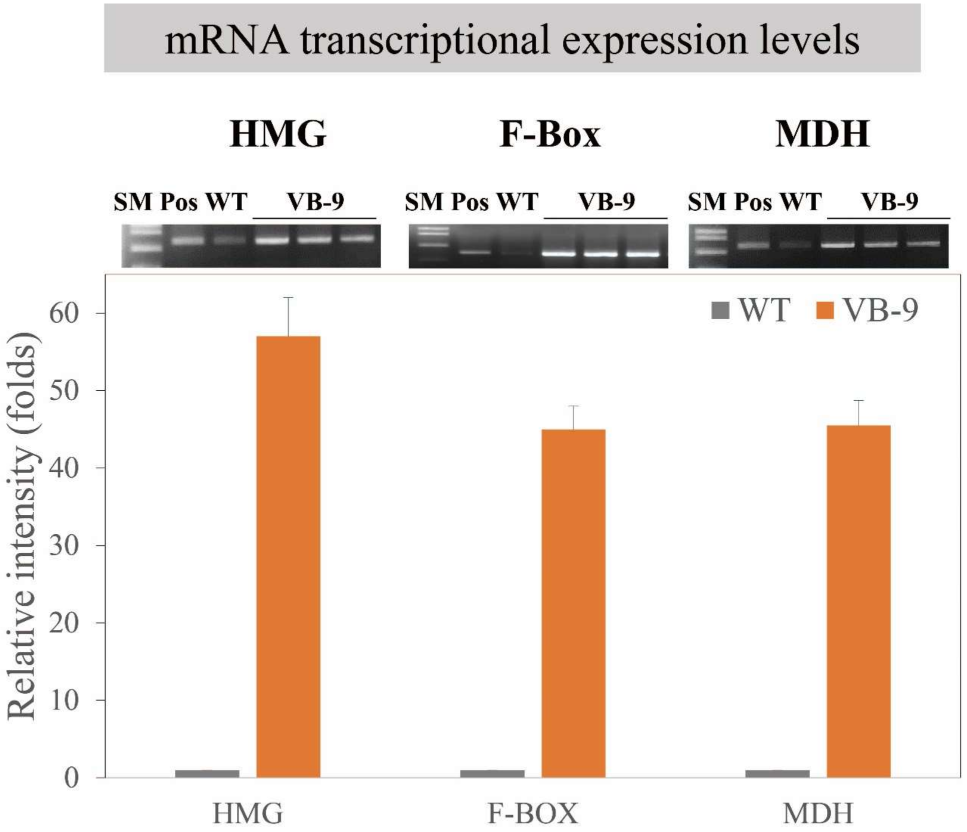

Supplemental Figure S3. Results of mRNA expression levels of HMG, F-Box, and MDH in the transgenic tobacco plants overexpressing *T. maritima* BglB targeted to the vacuole in comparison with those in the wild-type tobacco plants.

References

1. Wang, Y.; Fan, C.; Hu, H.; Li, Y.; Sun, D.; Wang, Y.; Peng, L. Genetic modification of plant cell walls to enhance biomass yield and biofuel production in bioenergy crops. *Biotech. Adv.* **2016**, *34*, 997–1017.
2. Lindén, P.; Keech, O.; Stenlund, H.; Gardeström, P.; Moritz, T. Reduced mitochondrial malate dehydrogenase activity has a strong effect on photorespiratory metabolism as revealed by 13C labelling. *Jour. Exp. Bot.* **2016**, *67*, 3123–3135.
3. van der Merwe, M.J.; Osorio, S.; Moritz, T.; Nunes-Nesi, A.; Fernie, A.R. Decreased mitochondrial activities of malate dehydrogenase and fumarase in tomato lead to altered root growth and architecture via diverse mechanisms. *Plant. Physio.* **2009**, 149, 653–669.
4. Tesfaye, M.; Temple, S.J.; Allan, D.L.; Vance, C.P.; Samac, D.A. Overexpression of Malate Dehydrogenase in Transgenic Alfalfa Enhances Organic Acid Synthesis and Confers Tolerance to Aluminum. *Plant. Physio.* **2001**, *127*, 1836–1844.
5. Li, Q.-F.; Zhao, J.; Zhang, J.; Dai, Z.-H.; Zhang, L.-G. Ectopic Expression of the Chinese Cabbage Malate Dehydrogenase Gene Promotes Growth and Aluminum Resistance in Arabidopsis. *Front. Plant. Sci.* **2016**, *7*, 1180–1191.
6. Wang, Q.F.; Zhao, Y.; Yi, Q.; Li, K.Z.; Yu, Y.X.; Chen, L.M. Overexpression of malate dehydrogenase in transgenic tobacco leaves: Enhanced malate synthesis and augmented Al-resistance. *Act. Phys. Plan.* **2010**, *32*, 1209–1220.
